# Nutritional Modulation of the Gut–Kidney Axis

**DOI:** 10.3390/nu18020263

**Published:** 2026-01-14

**Authors:** Razvan George Bogdan, Felicia Gabriela Gligor, Paula Anderco, Livia Mirela Popa, Adriana Popescu, Vlad Adam Bloanca, Elisa Leonte, Mihai Iliescu Glaja, Zorin Petrisor Crainiceanu, Cristian Ichim

**Affiliations:** 1 Faculty of Medicine, “Victor Babes” University of Medicine and Pharmacy, 300041 Timisoara, Romania; razvan.bogdan@umft.ro (R.G.B.); bloanca.vlad@umft.ro (V.A.B.); elisa.leonte@umft.ro (E.L.); mihai.iliescu.glaja@umft.ro (M.I.G.); crainiceanu.zorin@umft.ro (Z.P.C.); 2 Faculty of Medicine, Lucian Blaga University of Sibiu, 550169 Sibiu, Romania; felicia.gligor@ulbsibiu.ro (F.G.G.); adriana.popescu@ulbsibiu.ro (A.P.); cristian.ichim@ulbsibiu.ro (C.I.)

**Keywords:** gut–kidney axis, immunonutrition, chronic kidney disease, short-chain fatty acids, bioactive peptides, omega-3 fatty acids

## Abstract

Background: Chronic kidney disease (CKD) represents a state of persistent, sterile low-grade inflammation in which sustained innate immune activation accelerates renal decline and cardiovascular complications. Diet-induced gut dysbiosis and intestinal barrier dysfunction lower mucosal immune tolerance, promote metabolic endotoxemia, and position the gut as an upstream modulator of systemic inflammatory signaling along the gut–kidney axis. Scope: Most studies address microbiota-derived metabolites, food-derived bioactive peptides, or omega-3 fatty acids separately. This review integrates evidence across these domains and examines their convergent actions on epithelial barrier integrity, immune polarization, oxidative-inflammatory stress, and inflammasome-dependent pathways relevant to CKD progression. Key mechanisms: CKD-associated dysbiosis is characterized by reduced short-chain fatty acid (SCFA) production and increased generation and accumulation of uremic toxins and co-metabolites, including indoxyl sulfate, p-cresyl sulfate, trimethylamine *N*-oxide, and altered bile acids. Reduced SCFA availability weakens tight junction-dependent barrier function and regulatory immune programs, favoring Th17-skewed inflammation and endotoxin translocation. Bioactive peptides modulate inflammatory mediator networks and barrier-related pathways through effects on NF-κB/MAPK signaling and redox balance, while omega-3 fatty acids and specialized pro-resolving mediators support resolution-phase immune responses. Across these modalities, shared control points include barrier integrity, metabolic endotoxemia, oxidative stress, and NLRP3 inflammasome activation. Conclusions: Although evidence remains heterogeneous and largely preclinical, combined nutritional modulation targeting these convergent pathways may offer greater immunomodulatory benefit than isolated interventions. Future multi-omics-guided, factorial trials are required to define responder phenotypes and translate precision immunonutrition strategies into clinical CKD care.

## 1. Introduction

Within biomedical science, inflammatory activity is increasingly viewed as a temporal spectrum rather than a single uniform process. At one end lies acute inflammation, a short-lived, tightly regulated response that is particularly well described in the context of bacterial infection [1]. When microbes enter the host, they release endo- or exotoxins that trigger innate immune sensors, prompting immune cells to secrete inflammatory cytokines and chemokines [2]. These mediators attract leukocytes and activate complement at the affected site, where recruited cells generate proteolytic enzymes and reactive oxygen species to neutralize and clear the invading pathogens [3,4]. In this setting, the inflammatory cascade is self-limited and ultimately serves a protective role by restoring tissue integrity and preserving homeostasis [5].

At the opposite end of the spectrum is chronic inflammation (CI), which arises when the eliciting factor cannot be eradicated within a relatively short timeframe [6]. Under these conditions, immune cells remain continuously stimulated and maintain an ongoing production of inflammatory cytokines, driving a persistent inflammatory state throughout the organism [7]. Sustained activation of these pathways is thought to impose a substantial metabolic burden, with increased protein turnover and energy expenditure contributing to progressive functional decline [8]. Importantly, CI is not restricted to unresolved infections; dysregulated metabolism and persistent environmental exposures can also perpetuate inflammatory signaling [9,10,11].

In recent years, this conceptual spectrum from acute to CI has been increasingly applied to non-communicable diseases, including cardiometabolic and renal disorders, in which low-grade but persistent inflammatory activity amplifies tissue injury and accelerates organ failure [12,13,14]. Chronic kidney disease (CKD) exemplifies this paradigm, representing a state of sterile, non-infectious inflammation closely linked to cardiovascular morbidity. Sterile activation of the innate immune system is now recognized as a major pathogenic driver of CKD onset and progression and of its association with atherosclerotic cardiovascular disease (ASCVD), with central involvement of inflammasome-dependent pathways such as NLRP3 [15,16]. Together, these observations support the view that CKD is not only a state of reduced glomerular filtration but also a prototypical model of chronic, sterile inflammation.

Modern dietary patterns rich in ultra-processed foods can disrupt gut microbial ecology and weaken mucosal immune tolerance, thereby lowering the threshold for chronic low-grade inflammation [17,18,19,20,21,22]. Through diet-driven dysbiosis and barrier impairment, the gut becomes an upstream modulator of systemic inflammatory signaling relevant to cardio–renal disease.

The intestine represents a central immunometabolic interface, hosting a dense microbiota and a specialized epithelial barrier that separates the luminal environment from the internal milieu [23,24]. Along the gastrointestinal tract, bacterial load and composition are spatially stratified as follows: the stomach/proximal small intestine hosts low-density communities enriched for facultative anaerobes, whereas the colon contains the highest biomass and is dominated by obligate anaerobes within Firmicutes and Bacteroidetes [25]. Across the lifespan, the gut microbiome matures rapidly in early life (first ~3 years) and later remodels in older age, often with reduced diversity and relative expansion of Proteobacteria [26,27,28].

In CKD, gut dysbiosis, impaired barrier function, and increased intestinal permeability are frequently observed, together with the passage of bacterial products such as lipopolysaccharide into the circulation [29]. This state of metabolic endotoxemia sustains low-grade activation of innate immune receptors, amplifies systemic inflammation, and contributes to the progression of renal and cardiovascular injury [29,30]. These interactions form the basis of the gut–kidney axis concept, positioning the intestine as an upstream modulator of renal inflammation.

However, the existing literature typically addresses microbiota-derived metabolites, bioactive peptides, or omega-3 fatty acids in isolation. An integrated framework that considers their convergent actions on epithelial barrier integrity, metabolic endotoxemia, immune polarization, and inflammasome activation remains insufficiently developed. Accordingly, this review synthesizes current evidence on how microbial metabolites, food-derived bioactive peptides, and omega-3-derived specialized pro-resolving mediators can be combined within a precision immunonutrition framework to modulate the gut–immune–kidney axis in CKD.

## 2. Search Strategy

A targeted yet comprehensive literature search was conducted to identify experimental and clinical studies addressing the interplay between chronic low-grade inflammation, the gut–immune axis, and dietary modulators such as microbial metabolites, bioactive peptides, and omega-3 fatty acids. The search covered the literature up to December 2025, with earlier publications included when judged mechanistically or historically relevant. The main electronic database used was PubMed, complemented by Web of Science and Scopus to capture additional experimental and translational work. Search terms combined controlled vocabulary and free-text keywords grouped into four core domains as follows:

- Chronic inflammation and immune dysregulation (“chronic inflammation”, “low-grade inflammation”, “systemic inflammation”, “innate immunity”, “NLRP3 inflammasome”, and “intestinal permeability”);
- Gut–immune/gut–kidney axis (“gut–immune axis”, “gut–kidney axis”, “intestinal microbiota”, “microbiome-derived metabolites”, “dysbiosis”, “metabolic endotoxemia”, and “lipopolysaccharide”);
- Microbial metabolites and bioactive peptides (“short-chain fatty acids”, “SCFA”, “acetate”, “propionate”, “butyrate”, “bioactive peptides”, “food-derived peptides”, and “protein hydrolysates”);
- Omega-3 fatty acids and related lipid mediators (“omega-3 fatty acids”, “DHA”, “specialized pro-resolving mediators”, and “resolvins”).

The search was restricted to peer-reviewed articles published in English. Eligible studies included mechanistic in vitro work, animal models, human observational cohorts, interventional trials, and high-quality systematic reviews or meta-analyses. Abstracts, conference proceedings without full text, narrative commentaries lacking mechanistic depth, and articles not reporting any immune, inflammatory, or barrier-related outcomes were excluded.

## 3. Dietary Immunomodulators in the Gut–Kidney Axis

### 3.1. Gut Dysbiosis and Diet-Dependent Microbial Metabolic Remodeling in CKD

Under physiological conditions, the intestinal microbiota contribute fundamentally to host homeostasis across multiple organ systems by shaping metabolic activity, maintaining epithelial barrier integrity, and fine-tuning innate and adaptive immune responses, a balanced state commonly referred to as “symbiosis” [31]. By contrast, a community structure that promotes host damage has been termed “dysbiosis” [32].

In patients with CKD, gut dysbiosis was first characterized in a systematic manner in 2013 and has since been consistently confirmed and refined by studies using both 16S rRNA gene profiling and shotgun metagenomic sequencing [33,34]. These analyses demonstrate profound alterations in gut microbial ecology, with expansion of pathobionts (commensals that become harmful when overrepresented), depletion of beneficial commensal taxa and their metabolites, and a global reduction in microbial diversity, disturbances that mirror those observed in other chronic inflammatory and metabolic disorders [35,36,37,38].

In CKD cohorts, these alterations typically manifest as reduced α-diversity, overgrowth of Enterobacteriaceae as a prototypical pathobiont group, and a decline in commensal Firmicutes at phylum level [39,40,41,42]. The drivers of this dysbiotic state are incompletely understood but likely include low-fiber dietary patterns, muscle wasting, polypharmacy, uremic toxin accumulation, and constipation, all of which favor proteolytic fermentation over saccharolytic metabolism [43]. Beyond taxonomic shifts, CKD-associated dysbiosis is increasingly recognized as a state of functional remodeling, in which microbial metabolic output is redirected toward pathways that promote systemic inflammation and host injury.

In parallel with the reduction in saccharolytic fermentation and short-chain fatty acid (SCFA) production, CKD-associated dysbiosis is characterized by a shift toward proteolytic and choline- and carnitine-dependent microbial metabolism, resulting in increased generation and systemic accumulation of microbiota-derived uremic toxins and other pro-inflammatory co-metabolites [44,45].

Among the most extensively studied compounds are indoxyl sulfate (IS) and p-cresyl sulfate (p-CS), which originate from bacterial metabolism of dietary tryptophan and tyrosine/phenylalanine, respectively, followed by hepatic sulfation [46,47]. In CKD, impaired renal clearance combined with sustained intestinal overproduction leads to marked elevations of circulating IS and p-CS, which have been consistently associated with endothelial dysfunction, oxidative stress, vascular calcification, and accelerated progression of renal and cardiovascular disease [48,49,50].

Importantly, these solutes function as biologically active signaling molecules rather than inert retention products, activating redox-sensitive pathways and NF-κB–dependent inflammatory programs in renal tubular, endothelial, and immune cells, thereby reinforcing the chronic inflammatory milieu characteristic of CKD [51,52].

Trimethylamine *N*-oxide (TMAO) represents another diet–microbiome–host co-metabolite of relevance to the gut–kidney axis. TMAO is generated when gut microbiota convert dietary choline, phosphatidylcholine, and L-carnitine into trimethylamine (TMA), which is subsequently oxidized to TMAO in the liver [53,54,55]. Elevated circulating TMAO concentrations have been reported in patients with CKD and have been associated with increased cardiovascular risk and adverse outcomes, potentially through effects on endothelial function, platelet reactivity, lipid metabolism, and inflammatory signaling [56,57,58]. Although the causal role of TMAO remains debated and may depend on dietary context and host factors, the uremic environment is characterized by reduced renal excretion and an altered microbial ecosystem that together favor TMAO accumulation and may amplify its downstream pathophysiological effects [59,60,61].

### 3.2. Short-Chain Fatty Acids as Regulators of Barrier Integrity and Immune Tolerance

SCFAs such as acetate, propionate, and butyrate are key microbial metabolites generated through colonic fermentation of dietary fiber and act as central mediators of microbiota–host crosstalk [62,63]. By engaging G-protein-coupled receptors including GPR41, GPR43, and, in the kidney, GPR109A and by inhibiting histone deacetylases, SCFAs modulate innate and adaptive immune responses, promote regulatory T-cell differentiation, and support epithelial barrier integrity [62,63,64,65].

In CKD, gut dysbiosis is typically accompanied by a decline in SCFA production, resulting in attenuation of these anti-inflammatory and barrier-protective effects [66]. At the immune level, SCFAs promote the expansion and function of regulatory T cells, thereby constraining the release of pro-inflammatory cytokines such as TNF-α and interleukin-6 [67,68]. More broadly, microbiota-derived SCFAs contribute to systemic immune homeostasis by regulating the balance between pro-inflammatory T helper 17 (Th17) cells and regulatory T cells (Tregs), a central axis in immune-mediated tissue injury [69,70].

In CKD, dysbiosis and reduced availability of immunoregulatory microbial metabolites shift this balance toward Th17 polarization and/or functional expansion, while impairing Treg differentiation and suppressive capacity, thereby amplifying IL-17-driven inflammatory programs [45,71,72,73]. This Th17/Treg imbalance is increasingly recognized as a mechanistic link between intestinal dysregulation and extraintestinal pathology, including renal inflammation, where Th17-associated pathways promote leukocyte recruitment, cytokine amplification, and tissue injury [74,75,76].

Clinical and experimental data indicate that reduced circulating and fecal SCFAs concentrations in CKD correlate with markers of renal dysfunction, including elevated serum creatinine and lower glomerular filtration rate and that restoration of SCFA availability can ameliorate kidney injury [77]. Mechanistically, SCFAs attenuate systemic inflammation by suppressing TNF-α and interleukin-6 production and by modulating intracellular signaling pathways and cellular energy metabolism in renal and immune cells [78].

In parallel, SCFAs reinforce intestinal barrier function by upregulating tight junction proteins, thereby limiting bacterial translocation and endotoxemia, a recognized driver of CKD progression [79]. Experimental models further show that SCFAs can mitigate mitochondrial oxidative stress, reduce renal fibrosis, and help preserve tubular architecture, highlighting their potential as modulators of kidney structural integrity [80]. Collectively, these pleiotropic actions suggest that restoring SCFA signaling represents a promising strategy to slow CKD progression and improve renal outcomes [81].

Dietary manipulation is a practical means of enhancing SCFA generation. High-fiber diets and prebiotic substrates such as resistant starch and inulin increase the abundance and activity of butyrate-producing bacteria, thereby strengthening gut barrier function and reducing endotoxemia [82,83]. In CKD populations, such interventions have been associated with lower inflammatory markers and improved metabolic profiles, supporting a beneficial impact on the gut–kidney axis [84,85]. Moreover, combining a low-protein diet with prebiotics appears to further attenuate the burden of uremic toxins while reinforcing microbiota-dependent renoprotective mechanisms [86].

Beyond diet and prebiotics, microbiota-targeted approaches such as fecal microbiota transplantation have been explored as a means to restore microbial diversity and SCFA production in CKD [63,87,88]. However, evidence remains early and heterogeneous, and FMT should currently be considered experimental rather than a routine adjunct in CKD care [89,90].

### 3.3. Intestinal Barrier Dysfunction and Metabolic Endotoxemia

CKD and uremia profoundly impair intestinal epithelial barrier integrity, giving rise to a “leaky gut” phenotype characterized by increased intestinal permeability [91]. Elevated luminal urea diffuses into the gut, where bacterial urease activity generates ammonia and ammonium hydroxide, leading to the disruption of tight junction proteins and epithelial injury [92,93].

As a consequence, translocation of endotoxins such as lipopolysaccharide and other microbial products into the systemic circulation is facilitated, promoting metabolic endotoxemia [45]. This persistent influx of microbial-derived inflammatory stimuli sustains low-grade systemic inflammation through activation of innate immune receptors and cytokine release, thereby exacerbating renal injury and accelerating CKD progression [94,95,96].

Experimental and clinical studies in CKD and ESRD consistently demonstrate altered tight junction expression, increased circulating endotoxin levels, and heightened inflammatory markers, underscoring intestinal barrier dysfunction as a central pathogenic component of the gut–kidney axis [97,98,99,100]. Consequently, the gut–immune–kidney axis should be viewed not only as a metabolic circuit, but also as an immunologic relay through which dysbiosis may exacerbate immune-mediated kidney disease phenotypes. In this context, interventions that restore epithelial integrity and reduce endotoxin translocation may help attenuate systemic inflammation in CKD.

### 3.4. Bile Acid Metabolism and Additional Microbiota-Related Signaling Pathways

Beyond SCFAs and classical uremic toxins, gut dysbiosis in CKD also alters bile acid metabolism. Primary bile acids that escape ileal reabsorption are transformed by intestinal bacteria into secondary bile acids, which act as signaling molecules through host receptors such as the farnesoid X receptor and the G protein-coupled bile acid receptor TGR5 [101,102]. These pathways regulate glucose and lipid metabolism, epithelial barrier integrity, and immune homeostasis [102,103]. Experimental and clinical data indicate that CKD is associated with quantitative and qualitative alterations in the bile acid pool, which may contribute to metabolic inflammation, impaired barrier function, and dysregulated immune signaling along the gut–kidney axis [104,105]. Collectively, these observations underscore that CKD is marked not only by depletion of protective microbial metabolites such as SCFAs but also by enrichment of pro-inflammatory and vasculotoxic co-metabolites, many of which are strongly diet-dependent and therefore represent actionable targets for nutritional intervention.

### 3.5. Food-Derived Bioactive Peptides

Over recent years, naturally derived bioactive ingredients have attracted increasing interest, particularly phenolic compounds and food-derived peptides with health-promoting potential [106,107]. BAPs can be liberated from a wide array of animal- and plant-based proteins, including soybean, cereal germ, potato, nuts, dairy, egg, and meat [108,109]. In their parent protein sequences, these fragments are usually cryptic, but they acquire biological activity following proteolysis by enzymatic, chemical, or microbial processes, with enzymatic hydrolysis generally regarded as the most efficient and controllable approach [106,107,110]. Once released, many of these low-molecular-weight peptides are readily absorbed across the intestinal epithelium, enter the circulation, and reach peripheral targets, supporting their in vivo bioavailability and physiological effects [110].

Food-derived peptides are now recognized as multifunctional components capable of contributing to disease prevention and health maintenance [111]. Reported activities include antihypertensive, antithrombotic, anticancer, antimicrobial, antioxidant, immunomodulatory, and opioid agonist or antagonist effects, underscoring their broad bioactivity profile [112]. These functions are highly context-dependent and can be shaped by multiple variables, such as the protein source, pre-treatment, the primary amino-acid structure, and overall residue composition, in addition to molecular size and charge topology, pH, processing conditions, and specific chemical or physical treatments [106,107,113].

BAPs act on multiple organs, including the cardiovascular system, bone, and gut, and may also support immune function, stress adaptation, and mood [114,115]. Their activity is shaped by protein source, hydrolysis conditions, and enzyme choice, while antioxidant, antimicrobial, and angiotensin-converting enzyme (ACE) inhibitory effects depend on peptide length, amino acid composition, sequence, and overall structure and charge [116,117,118].

Interestingly, protein hydrolysates from various sources often display stronger antioxidant activity than individual purified peptide fractions, suggesting synergistic interactions among peptide mixtures and other co-extracted components [119]. Antioxidant and immunomodulatory peptides have been identified across diverse food matrices, supporting the concept that dietary proteins are a broad reservoir of bioactive sequences [120,121,122,123,124,125,126,127,128,129,130].

Recent investigations have substantially expanded the repertoire of food-derived peptides with antioxidant, dipeptidyl peptidase IV-inhibitory, and ACE–inhibitory activities, many of which display convincing bioactivity in chemical assays and cell culture models [131,132,133,134,135]. Together, these findings reinforce the view that peptides released from diverse dietary proteins constitute a rich source of candidates for future nutritional and pharmacological strategies [135,136,137,138]. Selected examples of milk-derived peptides with antioxidant, DPP-IV-inhibitory, and ACE-inhibitory activities are summarized in Table 1 [131,132,133,134,136,137,138,139,140].

### 3.6. Omega-3 Fatty Acids and Specialized Pro-Resolving Mediators

Omega-3 fatty acids are integral components of a healthy diet and have been proposed as adjunctive therapy for the management of CKD-related complications, as they can favorably modulate atherogenic lipid profiles, oxidative stress, systemic inflammation, and arterial hypertension [141,142]. At the cellular level, they participate in eicosanoid biosynthesis, influence membrane structure and fluidity, modulate multiple metabolic signaling cascades, and regulate the expression of numerous genes [143].

Several investigations have shown that patients with CKD have substantially lower circulating omega-3 fatty acid levels than the general population, a finding that may reflect reduced dietary intake, persistent inflammation, malabsorption, and underlying metabolic disturbances [141,144,145]. In individuals receiving hemodialysis, the burden of oxidative stress is further increased and may compromise omega-3 bioavailability and accelerate their depletion [146].

However, the clinical evidence remains inconsistent. Despite growing scientific interest over recent decades, omega-3 fatty acid supplements are still not widely used in CKD care, likely because the therapeutic advantages remain insufficiently defined and data are heterogeneous. While some studies have documented reductions in inflammatory markers in hemodialysis patients receiving oral omega-3 preparations, others have failed to detect meaningful effects on inflammatory indices or clinical outcomes [142,147,148].

## 4. Convergent Pathways and Potential Synergy

The gut microbiota act as a metabolically active ecosystem that links diet to immune signaling and barrier function through a limited set of recurrent molecular pathways [149,150,151,152,153]. In CKD, these pathways become particularly relevant because dysbiosis and barrier failure can sustain systemic innate immune activation and thereby amplify cardio–renal inflammatory injury.

Diet is a major determinant of microbiota composition and function and thereby shapes the pool of microbiota-derived metabolites that influence host physiology [154,155,156]. Complex carbohydrates escaping digestion are fermented by gut bacteria into SCFAs, while the amino acid tryptophan is converted into indole and related derivatives and cholesterol-derived primary bile acids are transformed into more soluble secondary bile acids in the distal gut [157,158,159]. These metabolites act as key mediators of microbiota–host crosstalk. Altered levels of SCFAs, indoles and secondary bile acids have been linked to disturbed metabolic and inflammatory states, whereas restoring their balance can attenuate disease progression in experimental and clinical settings [160,161,162,163,164,165]. Although many of these bacterial metabolites have been carefully identified and quantified in humans, the mechanistic routes through which they modulate systemic immunity and organ-specific inflammation are only partially understood [166,167,168].

BAPs with immunomodulatory and anti-inflammatory properties have emerged as promising candidates to support intestinal barrier repair and dampen mucosal inflammation, offering a potential adjunct or alternative to conventional enteritis treatments [169]. In a dextran sodium sulfate-induced colitis model, the walnut-derived tripeptide leucine–proline–phenylalanine promoted epithelial barrier restitution, lowered pro-inflammatory cytokine levels, and reduced intestinal epithelial apoptosis, while also partially normalizing gut microbial composition at selected doses [170]. Similarly, a fish collagen-derived preparation shifted macrophages towards an anti-inflammatory, immunotolerant, and antioxidative phenotype via a mannose receptor-dependent mechanism and ameliorated experimental colitis [171]. Antimicrobial peptides can also contribute to mucosal protection: the proteolysis-resistant peptide R7I attenuated inflammatory mediator release, preserved tight junction integrity, and restored near-normal intestinal histology in murine models of bacterial enteritis, highlighting its potential as a lead compound for enteritis therapy [172].

Inflammatory mediators encompass a wide range of low-molecular-weight molecules released by activated cells or present in body fluids during inflammation, including prostaglandins, nitric oxide, interleukins, chemokines, and other cytokines that collectively orchestrate the inflammatory response [173]. Engagement of toll-like receptors on innate immune cells induces production of interleukin-6, TNF-α, and TGF-β, while activating downstream nuclear factor-κB and mitogen-activated protein kinase (MAPK) pathways that further amplify pro-inflammatory signaling [174,175]. Cytokines, broadly categorized into pro-inflammatory mediators such as interleukin-1β and TNF-α and anti-inflammatory mediators such as interleukin-10, play a pivotal role in orchestrating immune cell proliferation, differentiation, and the resolution of inflammatory responses [176,177].

Multiple studies indicate that BAPs can modulate these mediator networks. Egg white-derived peptides reduced TNF-α and interleukin-6 production and downregulated messenger RNA expression of TNF-α, interleukin-6, interleukin-17, interleukin-1β, interferon-γ, and monocyte chemoattractant protein-1 in murine colitis models [174]. Bovine bone gelatin peptides lowered lipopolysaccharide-induced secretion of interleukin-6, nitric oxide, and TNF-α in RAW264.7 macrophages, and alleviated dextran sodium sulfate-induced colitis in vivo [178]. In a lipopolysaccharide-induced pneumonia model, the synthetic peptide SET-M33, designed to target Gram-negative bacteria, markedly decreased pulmonary levels of pro-inflammatory cytokines such as keratinocyte-derived chemokine, macrophage inflammatory protein-1α, interferon-inducible protein-10, monocyte chemoattractant protein-1, and TNF-α [179].

**Figure 1:**
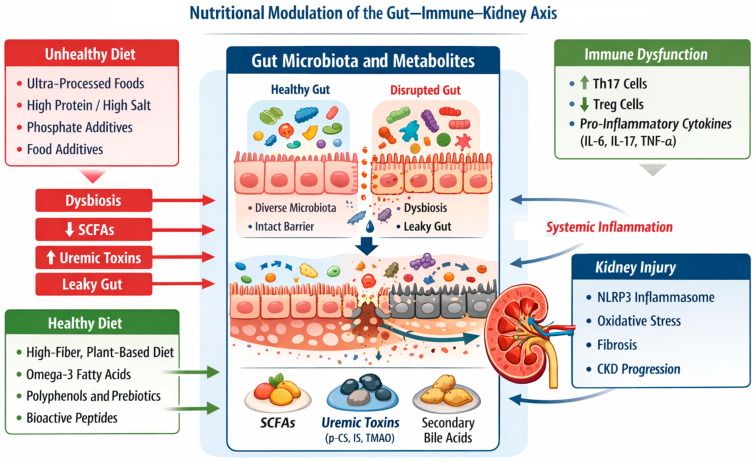
Nutritional modulation of the gut–immune–kidney axis. Schematic overview of the gut–immune–kidney axis in chronic kidney disease. Dietary patterns modulate gut microbiota composition and metabolic output, influencing intestinal barrier integrity, immune polarization, and renal inflammation. Dysbiosis-associated loss of beneficial metabolites and increased translocation of microbial products amplify chronic inflammation and kidney injury, while targeted nutritional strategies may restore microbial–immune homeostasis.

Although microbial metabolites, food-derived BAPs, and omega-3 fatty acids arise from distinct nutritional sources, their immunomodulatory actions cluster around a limited set of shared molecular hubs, particularly epithelial barrier integrity, metabolic endotoxemia, inflammasome activation, and the balance between inflammation-driving and inflammation-resolving immune programs in gut and kidney [180,181]. Figure 1 summarizes the convergent dietary, microbial, and immune pathways that link intestinal dysbiosis to systemic inflammation and renal injury in chronic kidney disease.

Oxidative stress is a key contributor to the decline in renal function in CKD, with higher stages of CKD showing increased reactive oxygen species (ROS) [182]. This excess arises from mechanisms such as mitochondrial dysfunction, enhanced nicotinamide adenine dinucleotide phosphate oxidase activity, and endothelial nitric oxide synthase uncoupling [2,182]. Oxidative stress and inflammation reinforce each other as follows: ROS amplify inflammatory cascades, while persistent inflammation further boosts ROS generation, sustaining a vicious cycle mediated by stress-activated kinases and redox-sensitive transcription factors [183,184,185,186]. Uremic toxins such as asymmetric dimethylarginine intensify this process by promoting ROS and reactive nitrogen species, which damage vascular structures, proteins, DNA, and mitochondria, activate NF-κB-driven cytokine production, and stimulate the renin–angiotensin system, thereby further increasing IL-6 and hepatic C-reactive protein synthesis [187,188,189,190]. Thus, oxidative stress is a central driver of CI in CKD rather than a secondary phenomenon [183].

### 4.1. Common Control Points: NLRP3 Inflammasome and Innate Immune Signaling

The NLRP3 inflammasome integrates danger signals derived from dysbiotic microbiota, oxidized lipids, uremic toxins, and metabolic stress, leading to caspase-1 activation and maturation of interleukin-1β and interleukin-18, which are key drivers of chronic kidney and cardiovascular injury [191]. Data from genetic mouse models indicate that NLRP3-dependent cytokine signaling actively contributes to both CKD progression and ASCVD, rather than merely reflecting secondary activation in advanced disease [192,193]. In parallel, interleukin-6, one of the most tightly regulated proinflammatory cytokines, has been implicated as a central mediator of systemic inflammation that fuels CKD progression and its cardiovascular complications, including ASCVD [194]. These observations underscore the direct pathogenic relevance of sustained innate immune activation and cytokine signaling to CKD progression and its cardio–renal complications.

SCFAs attenuate inflammasome activity primarily through G-protein-coupled receptors and histone deacetylase inhibition, which downregulate NF-κB-dependent priming and reduce NLRP3 activation in intestinal and extra-intestinal tissues [195,196]. In models of inflammatory bowel disease, SCFA supplementation reduces pro-inflammatory cytokines such as TNF-α and interleukin-1β, improves mucosal histology, and lowers systemic inflammatory markers, indirectly supporting reduced inflammasome activity in the gut–kidney axis [126,197,198].

Food-derived bioactive peptides influence the same molecular hubs predominantly at the level of NF-κB, MAPK, and redox balance, thereby modulating the priming step required for full NLRP3 inflammasome activation [169,199]. Experimental studies show that peptides generated from milk, egg, and plant proteins can suppress pro-inflammatory cytokines such as TNF-α and interleukin-6, reduce inducible nitric oxide synthase expression, and lower reactive oxygen species, all of which would be expected to constrain inflammasome activation in intestinal and renal tissues [173,200,201,202,203]. Beyond direct effects on immune cells, several bioactive peptides enhance tight junction protein expression and epithelial restitution, reducing translocation of pathogen-associated molecular patterns that otherwise sustain NLRP3 signaling [199,204].

Omega-3 polyunsaturated fatty acids and their specialized pro-resolving mediators (SPMs), including resolvins, target NLRP3 through complementary mechanisms, notably membrane re-organization, reduction in oxidative stress, and active promotion of resolution-phase programs [205,206,207]. In a spinal cord injury model, dietary omega-3 fatty acids reduced expression of NLRP3 components and downstream caspase-1 activation, illustrating inflammasome modulation by systemic omega-3 supplementation [205]. Aspirin-triggered resolvin D1, derived from docosahexaenoic acid, suppressed NLRP3 activation via autophagy-dependent mechanisms in neuropathic pain models, highlighting a direct link between omega-3-derived SPMs, inflammasome regulation, and tissue-specific inflammatory resolution [208].

### 4.2. Metabolic Endotoxemia and Epithelial Barrier Integrity

Low-grade metabolic endotoxemia, driven by increased intestinal permeability and translocation of lipopolysaccharide and other microbial products, has emerged as a key mechanism linking Western dietary patterns to systemic inflammation, metabolic syndrome, and chronic kidney disease [30]. Dysbiosis in chronic kidney disease is characterized by reduced SCFA-producing commensals and expansion of proteolytic and endotoxin-generating taxa, favoring luminal accumulation of uremic toxins and bacterial metabolites that impair gut barrier function [209,210]. SCFAs mitigate endotoxemia by supporting colonocyte metabolism and reinforcing tight junction-dependent barrier function, thereby reducing systemic exposure to microbial products [195,211].

Food-derived bioactive peptides add a second layer of protection at the epithelial interface. Several milk-, egg-, and plant-derived peptides reinforce tight junction organization, stimulate mucin secretion, and modulate innate immune receptors on epithelial cells, thereby attenuating barrier disruption in models of colitis and metabolic syndrome [169,199]. In addition, peptides with antioxidant and radical-scavenging properties decrease oxidative damage to epithelial and endothelial cells, which is an important cofactor in barrier failure and endotoxemia in both gut and kidney microvasculature [169].

Omega-3 fatty acids and their SPMs further stabilize barriers by modulating membrane composition, reducing endothelial activation, and promoting resolution of microvascular inflammation [206,212]. In experimental colitis, omega-3-derived resolvins reduce leukocyte adhesion, enhance epithelial restitution, and lower mucosal cytokine production, collectively limiting luminal–systemic molecular flux [180,206].

### 4.3. Organ-Specific Inflammatory Injury in Gut and Kidney

In the gastrointestinal tract, SCFAs have demonstrated therapeutic and immunologic benefits in models of inflammatory bowel disease, including reduced inflammation, lower expression of pro-inflammatory cytokines, and restoration of tight junction architecture [180]. These effects translate into decreased systemic inflammatory load and may indirectly mitigate kidney damage in settings where intestinal inflammation is a major contributor to the gut–kidney axis [213,214].

For omega-3-derived mediators, direct renal benefit has been demonstrated in experimental models. Resolvin D1 reduced tubular necrosis, inflammatory infiltrates, and serum cytokines in ischemia–reperfusion-triggered acute kidney injury by elevating regulatory T-cell frequencies via the ALX/FPR2 receptor pathway, suggesting an organ-specific mechanism of immune re-balancing [215]. In diabetic mice, the same mediator attenuated susceptibility to ischemic acute kidney injury by down-modulating NF-κB signaling and apoptosis, further supporting the concept that omega-3-derived SPMs can directly protect renal parenchyma under metabolic stress conditions [216].

## 5. Future Directions: Towards Precision Immunonutrition

Clinical data on SCFA-oriented interventions in CKD are still limited, but high-fiber, plant-forward dietary patterns and prebiotic supplementation have been associated with improved inflammatory profiles, reduced uremic toxin levels, and slower renal function decline in small trials and observational cohorts [217,218].

These observations align with the concept that diet is the most proximate upstream driver of the gut–kidney axis, with multiple nutrient classes exerting predictable effects on microbial ecology and host inflammation. First, higher intake of fermentable fiber and prebiotic substrates supports saccharolytic fermentation and SCFA production, and clinical trials/meta-analyses in CKD suggest this may reduce gut-derived uremic toxins and inflammatory markers [219,220]. Conversely, higher protein loads, particularly with lower fiber intake, can increase proteolytic fermentation and the generation of protein-bound uremic toxins, reinforcing systemic inflammation [221].

Beyond macronutrient balance, excess dietary sodium can also act as an immunomodulatory cofactor by reshaping the gut microbiome and promoting pro-inflammatory immune polarization (including Th17-associated programs), thereby potentially aggravating cardio–renal inflammatory stress [222,223]. In parallel, phosphorus intake (including inorganic phosphate additives) is clinically relevant in CKD and has been linked to measurable shifts in the intestinal microbiome in controlled dietary studies, supporting phosphorus management as part of a gut-aware renal nutrition strategy [224,225].

Finally, plant (poly)phenols may influence gut microbial composition and metabolite output, providing an additional lever to reduce oxidative-inflammatory signaling, while probiotics/synbiotics have been evaluated in randomized trials and meta-analyses with mixed but overall promising signals for improving inflammatory or uremic toxin-related endpoints [226,227].

However, these studies are heterogeneous in design, use different fiber sources and doses, and rarely include direct measurements of SCFA levels or microbiome composition, limiting mechanistic inferences. Selected recent mechanistic and clinical studies illustrating how microbial metabolites, bioactive peptides, and omega-3 fatty acids modulate the gut–immune axis and renal outcomes are summarized in Table 2.

Evidence for bioactive peptide-rich foods or peptide supplements in human gastro–renal disease is even more preliminary. Most data derive from animal models or early-phase studies focusing on blood pressure, vascular function, or surrogate inflammatory markers rather than hard renal or gastrointestinal endpoints [169,199,236]. The bioavailability and in vivo stability of specific peptides, their interaction with host proteases and microbiota, and inter-individual differences in digestion and absorption are major sources of variability that remain insufficiently characterized.

In parallel, antioxidant defenses are compromised. Vitamin D enhances nuclear factor erythroid 2-related factor 2 activity, upregulates antioxidant enzymes, and downregulates redox-sensitive inflammatory genes, but deficiency is frequent in CKD, blunting these protective effects [237,238]. Supplementation with vitamin D analogs, such as paricalcitol, has been associated with reduced C-reactive protein levels, suggesting a modulatory effect on systemic inflammation [239]. Serum albumin, the principal antioxidant protein in the circulation, is also depleted in CKD due to oxidative consumption and additional factors such as dietary restriction, impaired absorption, and diuretic use, which together lower plasma albumin concentrations [240]. In selected patients, albumin repletion may help to attenuate systemic low-grade inflammation by partially restoring antioxidant capacity [240].

For omega-3 fatty acids, several trials in CKD and dialysis populations have examined effects on cardiovascular risk factors, inflammation, and renal function, with mixed results that likely reflect differences in baseline diet, dose, formulation, and outcome selection [141,142,241]. Most studies have not stratified patients by inflammatory phenotype, microbiota composition, or genetic variation in fatty acid metabolism, which are crucial determinants of response and may partly explain neutral findings [242,243,244,245].

Precision nutrition frameworks in CKD propose integrating clinical, biochemical, and omics-based biomarkers to move beyond “one-size-fits-all” dietary prescriptions, aligning interventions with individual metabolic, inflammatory, and microbiome profiles [246]. While the concept of personalized or precision dietary modulation of the gut–kidney axis is conceptually attractive, its translation into routine clinical practice remains challenging. Inter-individual variability in CKD phenotype and stage, comorbid metabolic and cardiovascular disease, medication burden (including antibiotics, phosphate binders, iron preparations, and immunosuppressive agents), baseline dietary habits, and environmental exposures all shape gut microbiota composition and metabolic output, thereby influencing responsiveness to nutritional interventions [225,247,248,249].

In addition, host genetic factors, residual renal function, and dialysis modality further modulate immune and metabolic responses, limiting the applicability of uniform dietary prescriptions. These sources of heterogeneity likely contribute to the inconsistent results observed across clinical trials and underscore the need for stratified or biomarker-guided approaches rather than one-size-fits-all recommendations [246,250,251]. Accordingly, personalized nutrition in CKD should be viewed as a dynamic, iterative process that integrates clinical context, dietary feasibility, and emerging microbiome- and metabolite-based markers, rather than a fixed algorithmic intervention.

Within this paradigm, microbial metabolites, bioactive peptides, and omega-3 fatty acids can be viewed as modular levers that can be combined and titrated according to dominant pathophysiological axes in a given patient, such as barrier dysfunction, inflammasome activation, or oxidative stress [105,252].

From a practical perspective, a precision immunonutrition strategy for gastro–renal disease could include

- a high-fiber, plant-rich base diet to restore SCFA production;
- targeted use of prebiotics and microbiota-directed foods (including fermented, peptide-enriched products);
- personalized omega-3 supplementation optimized for dose, EPA/DHA ratio, and timing relative to inflammatory flares [199,218,253,254,255].

Emerging multi-omics studies indicate that diet-induced changes in microbiota-derived metabolites, including SCFAs, indoles, and secondary bile acids, can be mapped onto systemic inflammatory signatures and organ-specific outcomes, providing a framework for iterative refinement of such combined interventions [251,256,257]. Key methodological priorities include designing trials that incorporate deep phenotyping of the gut microbiome, metabolome, inflammatory mediators, and renal function, which explicitly test factorial combinations of fiber, peptide-rich foods, and omega-3 supplementation rather than evaluating each component in isolation.

Attention to inter-individual variability, arising from genetics, comorbidities, medication use, and baseline diet, will be essential to identify responder subgroups and build predictive models that can be translated into clinical decision tools for nephrology and gastroenterology practice.

Aging is a major, clinically relevant determinant of gut–kidney axis biology, because the gut microbiome changes across the lifespan and older adults tend to exhibit reduced microbial diversity, depletion of beneficial SCFA-producing taxa, and enrichment of pro-inflammatory pathobionts [258]. These age-associated shifts are frequently accompanied by impaired epithelial barrier resilience and “inflammaging”, a chronic low-grade inflammatory state driven by immunosenescence and sustained innate immune activation [259]. In CKD, where the disease burden is concentrated in older populations, age-related microbiome remodeling can therefore amplify dysbiosis, metabolic endotoxemia, and immune dysregulation, and may partly explain variability in response to dietary or microbiome-targeted interventions [260]. Accordingly, future trials should consider age and frailty/sarcopenia where relevant, as a stratification factor, and incorporate age-sensitive microbiome and metabolite endpoints to improve clinical translatability.

## 6. Conclusions

Chronic kidney disease exemplifies a state of persistent low-grade inflammation in which dysbiosis, barrier failure, and innate immune activation converge along the gut–kidney axis. In this context, microbiota-derived metabolites, food-derived bioactive peptides, and omega-3 fatty acids represent complementary nutritional levers that act on shared molecular hubs, including epithelial integrity, metabolic endotoxemia, redox balance, and NLRP3 inflammasome signaling.

Current evidence, although still fragmented and largely preclinical, supports the view that stacking these interventions by combining high-fiber, plant-rich dietary patterns that restore SCFA production with peptide-enriched foods and tailored omega-3 supplementation, may achieve more robust immunomodulatory effects than any single component alone. Future multi-omics-guided, factorial intervention trials in CKD are needed to disentangle causal pathways, define responder phenotypes, and translate this integrative framework into practical precision immunonutrition strategies for nephrology and gastroenterology practice.

## Figures and Tables

**Table 1:** Selected milk-derived BAPs with antioxidant, DPP-IV-inhibitory, and ACE-inhibitory activities, with potential relevance to cardio–metabolic and gastro–renal pathways. (Abbreviations: ACE: angiotensin-converting enzyme, DPP-IV: dipeptidyl peptidase IV, IC_50_: half-maximal inhibitory concentration, μM: micromolar, mM: millimolar, H_2_O_2_: hydrogen peroxide, C2C12: mouse myoblast cell line, and HepG2: human hepatocellular carcinoma cell line).

Source/Matrix	Peptide(s)	Primary Bioactivity	Experimental Model/Key Finding
Antioxidant peptides			
β-lactoglobulin (bovine)	ALPM, AVEGPK	Antioxidant	Reduced free radical formation in C2C12 myotubes (5 mM); ALPM protected HepG2 cells against oxidative injury
β-lactoglobulin (HepG2)	PKYPVEPF, LEASPEVI, YPFPGPIHNS	Antioxidant	Strong antioxidant activity in vitro; markedly improved survival of HepG2 cells exposed to H_2_O_2_-induced oxidative stress
Donkey milk	EWFTFLKEAGQGAKDMWR, GQGAKDMWR	Antioxidant	Endogenous antioxidant peptides, structurally defined and functionally validated
Buffalo milk cheese	AYF, YPFPGPIPK	Antioxidant	Newly described antioxidant peptides in buffalo milk cheese
ACE-inhibitory peptides			
β-lactoglobulin	YPFPGPIH, LKNWGEGW, RELEEIR, HPHPHLS	ACE-inhibitory	Low IC_50_ values (109.5, 77.7, 196.6, and 64.30 μM, respectively), indicating high inhibitory potency
Donkey milk	REWFTFLK, MPFLKSPIVPF	ACE-inhibitory	Isolated and structurally characterized as angiotensin-converting enzyme-inhibitory peptides
Buffalo milk cheese	LRF, APFPEVFGK	ACE-inhibitory	Newly described angiotensin-converting enzyme-inhibitory peptides in buffalo milk cheese
In silico (docking)	CLSPLQFR, TLMPQWW, CLSPLQMR	ACE-inhibitory (in silico candidates)	Showed favorable binding profiles at the catalytic site of ACE in molecular docking analyses
DPP-IV-inhibitory peptides			
β-lactoglobulin (screening)	LPV, IPT, PPL, PPQ, APL, PPT, APF, PPF, HPI, APS	DPP-IV-inhibitory	Identified by peptide-array screening as novel dipeptidyl peptidase IV inhibitors
Camel milk (trypsin hydrolysate)	FQLGASPY, FLQY, ILDKEGIDY, ILELA, SPVVPF, LQALHQGQIV, LPVP, MPVQA, LLQLEAIR	DPP-IV-inhibitory	Identified as dipeptidyl peptidase IV-inhibitory sequences in camel milk hydrolysates
Metabolic/insulin-related peptides			
Goat milk casein	SDIPNPIGSE, NPWDQVKR, SLSSSEESITH, QEPVLGPVRGPFP	Insulin-sensitizing/metabolic	Improved indices of insulin resistance in experimental models

**Table 2:** Recent experimental and clinical studies on microbial metabolites, bioactive peptides, and omega-3 fatty acids in gut–immune and gastro–renal inflammation (Abbreviations: AhR: aryl hydrocarbon receptor, SCFAs: short-chain fatty acids, CKD: chronic kidney disease, AKI: acute kidney injury, DSS: dextran sodium sulfate, UC: ulcerative colitis, EPA: eicosapentaenoic acid, DPA: docosapentaenoic acid, DHA: docosahexaenoic acid, ALA: alpha-linolenic acid, MCT: medium-chain triglycerides, Treg: regulatory T cell, NF-κB: nuclear factor kappa B, MAPK: mitogen-activated protein kinase).

Category/Pathway	Model/Population	Intervention/Exposure	Key Findings	Reference
Microbial tryptophan catabolites and AhR signaling	Human intestinal and hepatic cell models, AhR reporter assays	Panel of gut microbial tryptophan catabolites (indole, skatole, indole-3 derivatives, and kynurenines)	Multiple microbial tryptophan catabolites act as AhR ligands, indicating that shifts in microbial tryptophan metabolism can directly modulate epithelial and hepatic immune signaling.	[228]
Fiber-directed microbial tryptophan metabolism	Defined three-species community, human fecal cultures, and gnotobiotic mice	Fermentable fiber (pectin) reshaping competition for tryptophan among gut bacteria	Fermentable fiber redirects microbial tryptophan catabolism away from indole towards indole-3-lactic and indole-3-propionic acids, reducing potentially harmful indole production, and enhancing barrier-protective metabolites.	[229]
Butyrate, Treg cells, and colitis	Mouse models of colitis, in vitro T-cell polarization	Sodium butyrate supplementation	Butyrate promotes colonic Foxp3^+^ regulatory T-cell differentiation and ameliorates experimental colitis, linking commensal butyrate production to mucosal immune tolerance.	[230]
SCFAs and CKD progression	54 CKD patients + mouse model of AKI-to-CKD transition	Fecal propionate/butyrate measurement; oral SCFA treatment in mice	Propionate and butyrate levels fall with CKD severity and SCFA supplementation in mice attenuates renal inflammation, fibrosis, and progression to CKD.	[78]
Butyrate in diabetic nephropathy	Mice with diabetic nephropathy	Oral sodium butyrate	Butyrate improves albuminuria and renal histology and modulates AMPK/SIRT1/PGC-1α and mitochondrial dynamics, reducing inflammation and fibrosis in diabetic kidneys.	[231]
SCFAs, obesity, and low-grade inflammation	C57BL/6 mice on high-fat diet	High-fat diet with added acetate, propionate, butyrate, or SCFA mix	Dietary SCFAs limit weight gain, improve lipid profile, and reduce inflammatory cytokines while reshaping gut microbiota towards a less obesogenic pattern.	[232]
CKD, gut microbiota, and microbiota-targeted therapy	CKD and ESKD patients; systematic review of observational and interventional studies	Diet, prebiotics, probiotics, and synbiotics	CKD is consistently associated with loss of SCFA-producing taxa and expansion of uremic toxin producers, while small trials suggest microbiota-directed therapies can lower toxin load and inflammation.	[233]
Fish collagen peptides and colitis	DSS-induced colitis in mice; human monocyte-derived macrophages	Oral bioactive fish collagen peptides	Fish collagen peptides reduce colitis severity, promote anti-inflammatory macrophage polarization, improve tight-junction integrity, and partially normalize gut microbiota.	[171]
Antimicrobial peptide R7I and enteritis	Salmonella-induced enteritis in mice	Oral, proteolysis-resistant peptide R7I	R7I lowers mucosal pro-inflammatory cytokines, preserves villus structure and tight junction proteins, and improves gut barrier function in bacterial enteritis.	[172]
Anti-inflammatory food-derived peptides (review)	In vitro, in vivo, and in silico peptide studies	Dairy, fish, plant, and by-product protein hydrolysates	Short, hydrophobic, and basic residue-rich peptides consistently suppress NF-κB/MAPK pathways and pro-inflammatory mediators, highlighting structural motifs for designing anti-inflammatory bioactive peptides.	[234]
Serum resolvin E1 and ulcerative colitis	51 patients with ulcerative colitis + 30 controls	Serum resolvin E1 measurement	RvE1 levels are modestly higher in UC than in controls but do not clearly distinguish active disease from remission, limiting their utility as a stand-alone activity biomarker.	[235]
Circulating marine *n*-3 PUFAs and incident CKD	19 population-based cohorts without CKD at baseline	Baseline EPA, DPA, DHA, ALA, and fish/ω-3 intake; prospective follow-up	Higher circulating marine *n*-3 PUFAs (especially DHA) are associated with lower risk of incident CKD and slower eGFR decline, whereas ALA shows no clear association.	[147]
Omega-3 supplements in CKD patients on hemodialysis	120 CKD patients undergoing hemodialysis (randomized trial)	3 × 1000 mg/day omega-3 capsules vs. 3 × 1000 mg/day MCT placebo for 2 months	Omega-3 supplementation in hemodialysis patients significantly lowers BUN and serum creatinine compared with placebo, without affecting serum Na, K, Ca, or P.	[148]

## Data Availability

No new data were created or analyzed in this study. Data sharing is not applicable to this article.
